# Relationships between Physical Activity Level and Pain in the Spanish Population: A Cross-Sectional Study

**DOI:** 10.3390/jpm12101591

**Published:** 2022-09-27

**Authors:** Ángel Denche-Zamorano, Juan Manuel Franco-García, Raquel Pastor-Cisneros, Diana Salas-Gómez, Daniel Collado-Mateo, Pedro Rufino Olivares, José Carmelo Adsuar

**Affiliations:** 1 Promoting a Healthy Society Research Group (PHeSO), Faculty of Sport Sciences, University of Extremadura, 10003 Caceres, Spain; 2 Health Economy Motricity and Education (HEME), Faculty of Sport Sciences, University of Extremadura, 10003 Caceres, Spain; 3 Escuelas Universitarias Gimbernat (EUG), Physiotherapy School Cantabria, Movement Analysis Laboratory, University of Cantabria, 39300 Torrelavega, Spain; 4 Centre for Sport Studies, Rey Juan Carlos University, Fuenlabrada, 28943 Madrid, Spain; 5 Faculty of Sport Sciences, Universidad de Huelva, 21007 Huelva, Spain; 6 Universidad Autonoma de Chile, Talca 3480094, Chile

**Keywords:** exercise, sedentary lifestyle, health, fitness, physical therapy, musculoskeletal pain

## Abstract

Introduction. One third of the world’s population suffers from some form of pain. Physical inactivity is one of the causes that reduces physical fitness and may lead to an increase in the prevalence of pain in the population. Aims. To analyse the relationships between the level of physical activity (PAL) and the prevalence and degree of pain, the limitations and impact of pain on daily activities and the use of pain medication in the Spanish population. Hypothesis. PAL is related to pain among Spaniards. Methodology. A cross-sectional study design was used, based on data obtained from the Spanish National Health Survey 2017 with 17,777 participants. A descriptive analysis was performed. Nonparametric statistical tests were used: chi-square statistic to analyse intergroup differences in ordinal variables; Mann–Whitney U test to analyse intergroup differences in continuous variables. A correlation study was also performed between the variables of interest, using Spearman’s rho. Results. Relationships were found between PAL and: prevalence of pain, degree of pain, limitations due to pain in usual activities, level of impact in daily activities and use of pain medication in the Spanish population (*p* < 0.001). Performing moderate and intense PA was related to lower prevalence and degree of pain in the population that performed it, compared to those who only walked or were inactive. Weak correlations were found between the level of PA and the study variables (*p* < 0.001). Conclusions. High PALs in the population are related to better indicators of pain among Spaniards, appearing to reduce the prevalence and degree of pain, as well as the limitations and impact caused by pain in the daily activities of citizens, and could reduce the use of pain medication in the adult Spanish population.

## 1. Introduction

One third of the world’s population suffers from some form of pain [1]. Pain has been defined by the International Association for the Study of Pain (IASP) as “an unpleasant sensory and emotional experience associated with actual or potential tissue damage, or described in terms of such damage” [2].

When pain is prolonged beyond the normal tissue healing time, it is considered chronic pain [3]. However, some authors suggest that this definition may not be the most accurate, considering that it may distract focus from the different factors that contribute to the pain experience, limit the patient’s understanding of their condition and the patient’s strategies to manage it, because currently, it is a term that is used when the causes of the pain have not been identified, considering that pain is the only medical problem to be treated [4,5]. Chronic pain reaching 1.5 billion people across the globe [6] has a high impact on quality of life, causing disability, contributing to increased symptoms of depression and anxiety, loss of work productivity [7] and sleep disorders [8,9] in those who suffer from it, as well as high health and social costs to society [10,11,12].

Physical inactivity and sedentary lifestyles are part of the global problems of today’s society, being one of the main concerns of governments and other world organisations. [13,14]. The World Health Organisation (WHO) defines physical inactivity and sedentary behaviour as “a level of physical activity (PAL) insufficient to meet current physical activity (PA) recommendations” and “any waking behaviour characterised by energy expenditure equal to or less than 1.5 METs” [15]. PA is defined by the WHO as “any bodily movement produced by skeletal muscles that requires energy expenditure” and can be performed at light, moderate or vigorous intensities [15]. In recent decades, lifestyle habits have evolved towards increasingly less active and more sedentary behaviour, leading to a reduction in physical fitness, as well as an increase in the prevalence of overweight and obesity in the global population [15]. Moreover, these behaviours form part of the main modifiable risk factors for the occurrence of cardiovascular diseases [16], different types of cancers [17], hypertension [18], type II diabetes, metabolic syndrome and other pathologies [19], as well as an increase in the prevalence of pain. In this sense, the WHO considers it a public health problem [3].

Relationships have been found between physical inactivity and an increased prevalence of different conditions and degrees of pain in all types of patients, with a positive relationship between greater physical activity and pain reduction, and increased physical inactivity and the occurrence of pain [20]. Conversely, a reduction in the prevalence of pain occurrence in general and a reduction in the degree of pain in pain sufferers have been found to be associated with increased PA [21]. The same has been found both in cases of people with chronic pain [20,22], and in different types of pain and types of patients: pain reduction in patients with fibromyalgia [23,24], with low back pain [25], with knee and/or hip osteoarthritis [26] or other types of pain in patients.

Therefore, the purpose of this study was to analyse the relationships between PAL and the prevalence of pain in the Spanish population, as well as to study the relationships between PAL and the degree of pain, the limitations caused by pain, the levels of pain impairment in daily activities and the use of pain medication in this population. Furthermore, to analyse these relationships in both sexes. The starting hypothesis of the present study was: PAL is related to pain in the Spanish population under 70 years of age; and PA of moderate and/or vigorous intensity is related to a reduction in the proportion of people with pain compared to just walking or being sedentary in this population.

## 2. Materials and Methods

### 2.1. Design

Cross-sectional descriptive and correlational study was conducted using the adult questionnaire from the Spanish National Health Survey 2017 (ENSE2017), based on data from the ENSE2017, the last major health survey conducted in Spain before the COVID-19 pandemic.

### 2.2. Participants

The 23,089 individuals, aged 15–103 years, were randomly selected after stratified three-phase sampling to take part in the ENSE2017 [27,28]. The ENSE is a five-yearly survey, conducted by the Ministry of Health, Consumer Affairs and Social Welfare (MSCBS) in collaboration with the National Institute of Statistics of Spain (INE). This survey “collects population-wide health information on health status, personal, social and environmental determinants of health, and use of and access to health services” [27] and was carried out between October 2016 and October 2017 by accredited interviewers.

A total of 10,595 men and 12,494 women were interviewed. For this study, participants over 69 years of age were excluded, as they were not questioned about their PA in the ENSE2017. The final sample was 17,777 participants, 8529 men and 9248 women.

### 2.3. Variables and Procedures

Based on data from the ENSE2017, the following variables were analysed:

Age. Collected in numerical values in the ENSE2017, it was used to characterise the sample. Of the group, 5312 participants were excluded from all analyses because they were over 69 years of age.

Sex. Used to characterise and group the sample into men and women.

Height and weight. Used to create the numerical body mass index (BMI) variable, based on the formula: weight (kg)/height (metres) squared. Here, 504 participants were discarded due to a lack of data on the variables weight (295) and height (209) for the characterisation of the population with this variable.

Limitation due to pain. Based on item Q.28, the population was grouped according to whether or not they had limitations in their usual activities during the last two weeks prior to the survey: “During the last 2 weeks, have you had to reduce or limit your usual activities for at least half of a day, because of one or more pain or symptoms?” With the following responses: “Yes” or “No”.

Degree of pain. The population was grouped according to their answers on item Q.45 in the ENSE2017: “During the last 4 weeks, what degree of pain have you experienced?”. With the following possible responses: none, very mild, mild, moderate, severe, extreme and don’t know or don’t answer (NS/NC). For the analyses with this variable and its derivatives, 4 participants were excluded for answering NS/NC.

Prevalence of pain. From item Q.45. A new variable was created to group the population according to whether or not they had experienced pain in the last 4 weeks: “Yes” (including those who responded that they had experienced some degree of pain); “No” (including those who responded “None”). For the analyses with this new variable, 4 participants were excluded for responding for answering NS/NC.

Level of pain effect. The population was grouped according to their answers on item Q.46 in the ENSE2017: “During the last 4 weeks, to what extent did pain affect your daily activities?”). With the following possible answers: not at all, a little, moderately, quite a lot, a lot and NS/NC, 4 participants were excluded for answering NS/NC for the analyses with this variable.

Pain medication. From item Q.85 in the ENSE2017: “During the last 2 weeks, have you taken any medication that was prescribed by a doctor?”, with possible answers: “Yes”; “No”; and item Q.87a.2: “Next I am going to read you a list of types of medication, please tell me which one(s) of them you have taken in the last 2 weeks? Pain medication?”) with possible responses: “Yes”, “No”, “NS/NC”; the population was grouped into those who did not take pain medication (answering “No” to both items, or “Yes” to item Q.85 and “No” to item Q.87a.2), and those who did take pain medication (answering “Yes” to both items), 1 participant was excluded for answering “No” to item Q.85.

Physical activity level (PAL). This was obtained from the data obtained in the questions of the Spanish short version of the International Physical Activity Questionnaire (IPAQ-SF). It asked about the frequency, duration and intensity of PA performed in the last 7 days (number of days and usual duration at each intensity). PAL groups were formed on the basis of a Physical Activity Index (PAI), calculated with the following formula [29,30]:

IAF = (intensity factor for vigorous activity * frequency factor for vigorous activity × duration factor for vigorous activity) + (intensity factor for moderate activity × frequency factor for moderate activity × duration factor for moderate activity).

The factors were:

Intensity factor: intensity factor for vigorous activity (10), intensity factor for moderate activity (5) and intensity factor for mild activity (0) [30]. These factors were applied to the following items: Q.113 and Q.115.

Frequency factor: to the questions, how many days did you do vigorous PA (Q.113) and how many days did you do moderate PA? (Q.115) The factors 0–3 were applied for the answer: “zero days” (0), “one day per week” (1), “two or three days per week” (2) and “more than three days per week” (3) [30].

Duration factor: to the questions “How much time did you spend in total on vigorous PA?” (Q.114) and “how much time did you spend in total on moderate PA?” (Q.116) The factors 1–1.5 were applied for the answer: “duration of less than 30 min” (1) and “duration of 30 min or more” (1.5) [30].

PAI values could range from 0 to 67.5. Mild activities did not add value to the PAI, only moderate and/or intense activities. Thus, six PALs were established, two of them, grouping people who reported no moderate and/or vigorous PA (“Inactive”: people with PAI = 0. In addition, to the question “Now think about how much time you spent walking in the last 7 days” (item Q.117 in the ENSE17), they answered “No day more than 10 min in a row”. “Walker”: People with PAI = 0. To the question, “Now think about how much time you spent walking in the last 7 days” (item Q.117 in ENSE17), declared to dedicate at least one day more than 10 min in a row) and four of them, corresponding to people who performed moderate/intense PA (“Low”: PAI = 1–15. “Medium”: PAI = 16–30. “High”: PAI = 31–45. “Very high”: PAI > 45).

For analyses including this variable, 60 participants were excluded for answering “NS/NC” to the frequency and duration of intense PA, items Q.113 (18) and Q.114 (13), and to the frequency and duration of moderate PA, items Q.115 (12) and Q.116 (17).

### 2.4. Statistical Analysis

The distribution followed by the different variables was studied, using the Kolmogorov–Smirnov normality test. Non-parametric tests were performed, due to not finding sufficient evidence to assume normal distributions for each of the variables. A descriptive analysis was performed to characterise the sample, presenting the variables age and BMI according to the central values: median and interquartile range.

The variables: degree of pain, prevalence of pain, pain limitation, level of pain impairment, and use of pain medication and level of PA were presented in absolute and relative frequencies. All of them, in the general population and by sex. A Mann–Whitney U test (for continuous variables), a chi-square test and a pairwise z-test for independent proportions (for ordinal variables) were performed to analyse associations between variables and differences between sexes and PAL groups.

Finally, Spearman correlation coefficients were used to evaluate the relationships between PAL and degree of pain, prevalence of pain, pain limitation, level of pain affect and use of pain medication, interpreting their results according to Mondragon [31].

The SPSS statistical package (version 25.0 SPSS Inc., Chicago, IL, USA) was used for the statistical analysis. The significance level for the analyses was established at *p* < 0.05.

## 3. Results

Table S1 shows the descriptive analysis to characterise the sample, as well as the dependence relationships between the categorical variables and sex, and the differences between the proportions of these between sexes. The median age of the sample was 47 years (21), in men and women, with no statistically significant differences, *p* = 0.295. The BMI of the sample had a median of 25.3 (5.9), being higher in men than in women (26.1 vs. 24.2, *p* < 0.001). Some 42.4% of the population reported having experienced some type of pain in the four weeks prior to the survey. The degree of pain was found to be related to sex (*p* < 0.001 from Chi-square test) with 35.3% of men reporting some kind of pain, compared to 48.9% of women, a difference of 13.6 percentage points (*p* < 0.05 from *z*-test). One in five Spaniards presented a degree of pain between moderate and extreme (20.2%), this being higher in women than men (25.3% vs. 15.3%, *p* < 0.05 from *z*-test). Furthermore, it was found that 30.3% of the Spanish population acknowledged feeling affected in their usual activities by pain between a little and a lot, this proportion being higher in women than men (36.0% vs. 24.6%, *p* < 0.05). A smaller proportion of people reported feeling limited by pain in their daily activities (13.4%), with a higher proportion of women than in men (15.5% vs. 11.1%, *p* < 0.05). The proportion of the Spanish population in this study who acknowledged taking pain medication reached 30.9%, being higher in women (37.5%) than men (23.7%), *p* < 0.001. PAL was found to be related to the sex of the participant (*p* < 0.001). Women were represented in higher proportions than men in the lower PAL groups.

**Figure 1:**
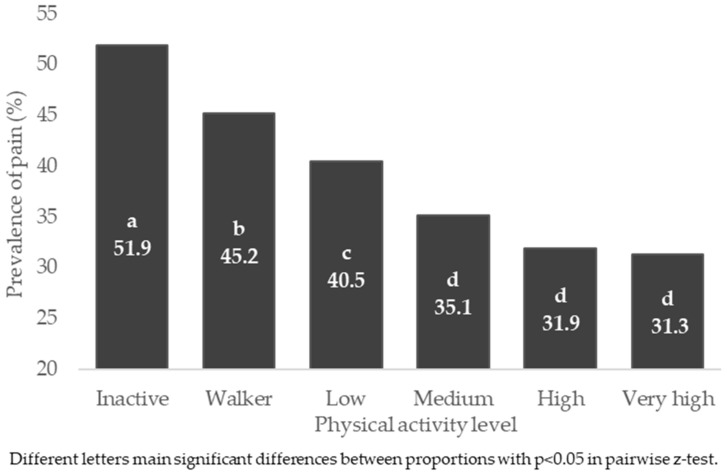
Prevalence of pain, according to physical activity level in general Spanish population.

The prevalence of pain was associated with the PAL in the general population and by sex, *p* < 0.001. The highest prevalence of pain was found in the inactive groups of the general population, men and women, being higher than walkers and other PALs (*p* < 0.05) (Table S2). Figure 1 shows the prevalence of pain in the general population, according to PAL of the participants.

The degree of pain was related to the PAL (*p* < 0.001). Extreme pain appeared in 3.4% of the inactive population, by proportions of less than 1% in people who performed moderate and/or vigorous activities, PALs equal, or higher than “Low”. Similarly, decreases were found in the proportions of people with “severe” and “moderate” degrees of pain as the PAL increased from “Inactive” to people with “Walker” PAL and from this level to higher levels, both in the general population and in men and women (Table S3).

**Figure 2:**
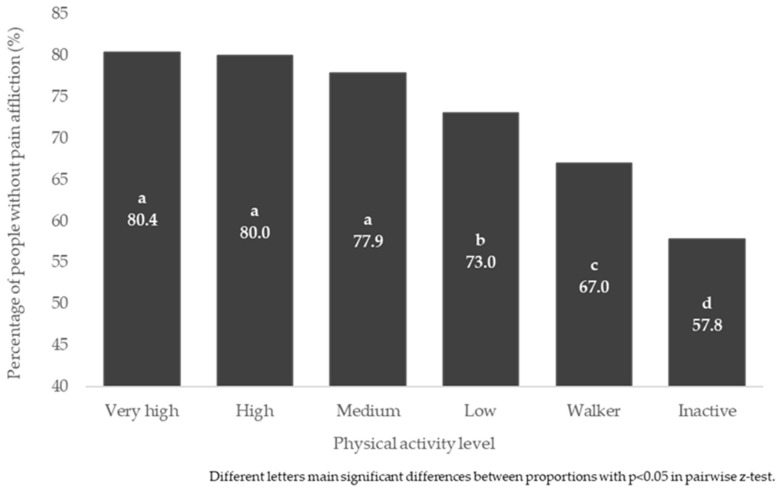
Percentage of people in the general population without pain effect, according to physical activity level.

Pain impairment in daily activities was found to be related to PAL in the general population and sexes, *p* < 0.001. The proportions of people not affected in their daily activities by pain were lower in “Inactive” than in the other PAL, *p* < 0.05. Figure 2 shows the proportions of people in the general population unaffected by pain in their daily activities, according to PAL. Differences in proportions were also found in both men and women similar to those found in the general population (*p* < 0.05) (Table S4).

Relationships was found between PAL and pain limitations in daily activities in the Spanish population, *p* < 0.001. These relationships were also found in both sexes, *p* < 0.001. More than one in five people in the “Inactive” group (21.5%) acknowledged that they were limited in their daily activities by pain and presented the highest proportions of pain limitations among all the PALs, *p* < 0.05. At the highest PAL in the general population, the proportions were more than halved: Low (10.7%), Medium (9.8%), High (8.5%) and Very high (9.4%) (Table S5).

**Figure 3:**
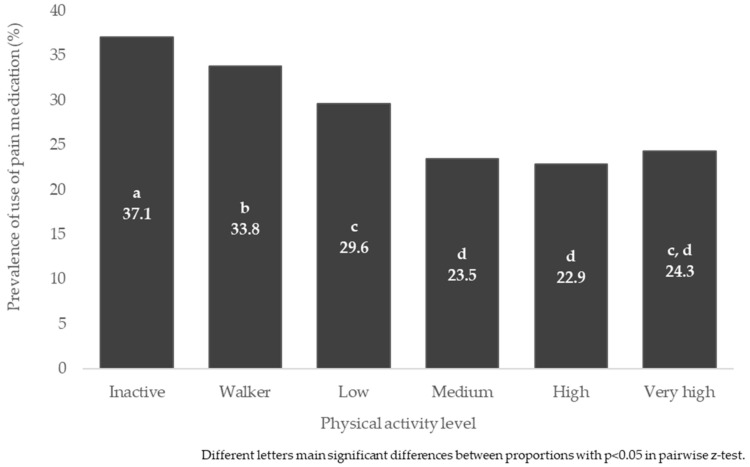
Prevalence of use of pain medication in the general population, according to physical activity level.

Finally, the use of pain medication was also found to be related to the PAL, *p* < 0.001 Medication use was found to be higher at lower PAL than at higher PAL. Figure 3 shows the prevalence of use of pain medication in the general population, according to PAL, with a total of 37.1% of inactive people reporting the use of pain medication, compared to proportions of 22.9, 23.5 and 24.3% in the “High”, “Medium” and “Very high” groups, respectively, *p* < 0.05) (Table S6).

Statistically significant weak negative correlations were found for Spearman’s rho between PAL and all pain-related variables investigated in this study (Table 1).

## 4. Discussion

The main purpose of this study was to analyse the relationships between PAL and the prevalence of pain in the Spanish population, as well as to study the relationships between PAL and the degree of pain, the limitations caused by pain, the levels of pain impairment in daily activities and the use of pain medication in this population.

Confirming the initial hypothesis, PAL in the Spanish population is related to all the analysed pain variables: degree, prevalence, limitation, level of pain impairment, and use of medication. These relationships were found in the general population and both sexes; pain prevalence was lower in the groups with high PAL, and higher in the inactive groups.

Another important finding was the existence of a relationship between moderate and/or vigorous PA with a higher proportion of people with a lower prevalence of pain, limitations in their usual activities due to pain and use of medication for pain, as well as lower degrees and levels of pain impairment in their daily activities compared to only walking and/or being sedentary. These results are partly similar to those reported in a study in the Dutch population (*n* = 3364), in which moderately physically active people were associated with a lower frequency of back pain than physically inactive people [32]. In addition, in our study, the group of people who walked at least one day a week for more than 10 min at a time showed lower proportions in the variables of interest compared to people with less physical activity.

Furthermore, the present study found relationships between the degree of pain and PAL, something that has already been evidenced in numerous studies with all types of people, different sexes and fitness levels, thanks to different mechanisms and factors [20,21,22,23,24,25]. In the general population, the prevalence of people without pain was 20 percentage points lower between inactive (48.1%) and the highest PALs (High: 68.1%; Very high). Conversely, the percentage of people with different degrees of pain decreased from the inactive group to those with moderate and/or intense PA. This was found in both sexes with statistically significant relationships. Only 42.8% of inactive women reported no pain, compared to 59.1% of women with a “High” activity level. In men, these percentages ranged from 54.3% inactive to 73.9% at the “Very high” level.

Some of these findings have been reported previously. For example, a study that examined the relationship between PA and musculoskeletal pain in adults over the age of 50 years in a cross-sectional analysis, reported that all levels of PA were associated with a lower risk of reporting musculoskeletal pain. On the other hand, several authors report a U-shaped relationship between PA and pain. In this U-shaped relationship, low values of PA (inactive) and high levels of physical activity are associated with a higher level of pain. Thus, they suggest that PA may be a risk or protective factor depending on the intensity. In this sense, some authors suggest that these different results depend on the statistical analysis and categorisation of the level of physical activity in some studies. It, therefore, seems important to have objective ways to quantify the level of physical activity prescribed to each patient or person.

Among the possible interventions to control the development or intensity of pain in the population, it is important to have a complementary non-pharmacological approach to pain management that targets modifiable risk factors that contribute to developing chronic pain such as physical inactivity [33,34]. Therefore, although more studies are needed to further investigate the relationship between physical activity levels and pain ratings, the available evidence suggests that physical activity and exercise is an intervention with few adverse effects that can improve pain severity and physical function, and consequently, quality of life [3].

Therefore, increasing the PAL of the Spanish population and/or implementing PA programs that incorporate moderate and vigorous PA could be advisable to reduce the proportion of people with pain, or reduce the degree of pain presented, thus improving the self-perceived health of the population. In this sense, current evidence suggests that a mere recommendation to increase physical activity may be insufficient to generate a significant change and may also lead to a higher percentage of adverse events [35,36]. It is essential to be able to adapt the physical activity to the conditions of the individual and his or her current state of health and fitness. Therefore, structured, guided physical activity programs with a progression of intensity individualised to each person may be more effective. In addition, physical activity plays an important role in improving and maintaining health by having positive effects on improving the overall physical and mental health, and physical functioning and decreasing the stress of the general population and people suffering from pain [3].

The second aim of this study was to analyse whether the relationship between PA and pain was different between sexes. A lower level of physical activity was detected in Spanish women in this study. Similar results have been reported in epidemiological studies in other countries, which show that women are generally less physically active compared to men [37]. The lower PA of women found in this study could have some relation with the higher degree of pain and the higher prevalence of pain found in women.

More than a third of the general Spanish population reported suffering from some type of pain compared to 57.6% who reported no pain. The degree of pain is related to sex (*p* < 0.001), being more present in women than in men (*p* < 0.05), with a difference of 13.6 points between men (64.7%) and women (51.1%) with no pain. In addition to a higher proportion of women with pain overall in this study, a higher proportion of women with pain in each category of pain degree was also detected. These gender differences have been widely reported and debated. The most common hypothesis is the structural and functional differences in the brain between men and women, as well as differences in pain processing pathways, hormonal influences in pain processing, genetic factors, or sociocultural and immunological differences which could at least partly explain the higher risk of women developing chronic pain [38,39,40]. However, despite this finding being reported in the literature, the possible mechanisms underlying gender differences in pain need to be further investigated.

This study has the inherent weaknesses of a cross-sectional design since reverse causality cannot be ruled out. Another limitation is that the differences between moderate and intense PA have not been analysed, a future aspect to be investigated that would allow us to assess whether there is a U-shaped relationship between PA and pain in the Spanish population, as has been previously reported in other populations. In addition, the way of quantifying the level of physical activity using indirect instruments could also be a limitation as several studies have reported that the IPAQ–SF overestimates PA level [41]; however, it has shown a good correlation with measurements in some populations [42]. Therefore, as mentioned above, it would be necessary to quantify BP directly and objectively to assess its relationship with pain in the population.

Another limitation is the lack of information that could be relevant to understanding the relationships found in this study, such as participant history, medical parameters, follow-up, and/or a 24 h compositional analysis. Finally, other sociodemographic variables that could condition the results, such as civil status, socio-economic status or level of education, were not taken into account in the present study. Future studies should examine the effects of these variables in studies assessing the relationship between PA and pain.

## 5. Conclusions

PAL is related to pain in the Spanish population. PAL was related to a lower prevalence of pain and lower degrees of pain in the Spanish population. Increasing PAL in the population could reduce the proportion of people with pain and the degree of pain present in the population.

High levels of PA in the population are related to lower proportions of people with limitations due to pain in their daily activities and with a lower level of pain impairment in these activities than in groups with lower PALs and/or inactivity.

The use of pain medication is related to PAL. Population groups with higher PALs have lower proportions of pain medication use than those who only walked and those who were inactive.

Moderate and intense PA appears to reduce the proportion of people with pain in the PA population groups who do moderate and intense PA compared to those who only walk. Walking could be an alternative, when higher intensity activities are not possible, when the aim is to lower the percentage of the population with pain, reduce the proportion of people with high levels of pain, or reduce limitations, the degree of pain effect in daily activities or reduce the use of pain medication.

## Figures and Tables

**Table 1:** Relationships between level of physical activity and prevalence of pain, degree of pain, pain limitation, level of pain impairment and use of pain medication in the Spanish population in 2017.

Physical Activity Level						
Variables	Total		Men		Women	
	Rho	*p*	Rho	*p*	Rho	*p*
Prevalence of pain	−0.125	<0.001	−0.133	<0.001	−0.088	<0.001
Degree of pain	−0.151	<0.001	−0.154	<0.001	−0.120	<0.001
Pain limitation	−0.107	<0.001	−0.101	<0.001	−0.102	<0.001
Pain affliction	−0.164	<0.001	−0.171	<0.001	−0.134	<0.001
Use of pain medication	−0.063	<0.001	−0.037	0.012	−0.067	<0.001

Rho (Spearman’s correlation coefficient with Bonferroni correction factor); *p* (*p*-value).

## Data Availability

https://www.sanidad.gob.es/estadEstudios/estadisticas/solicitud.htm; Accessed on 21 September 2022.

## Supplementary Materials

The following supporting information can be downloaded at: https://www.mdpi.com/article/10.3390/jpm12101591/s1, Table S1: Descriptive analysis of the Spanish population from the Spanish National Health Survey 2017; Table S2: Relationship between level of physical activity and prevalence of pain in the Spanish population; Table S3: Relationship between level of physical activity and prevalence of pain in the Spanish population; Table S4: Relationship between level of physical activity and prevalence of pain in the Spanish population; Table S5: Relationship between level of physical activity and suffering limitations to perform daily activities due to pain in the Spanish population of the ENSE 2017; Table S6: Relationship between level of physical activity and the use of pain medication in the Spanish population in the ENSE 2017.
