# Supplementary material for: Relationships between Physical Activity Level and Pain in the Spanish Population: A Cross-Sectional Study
Source: J Pers Med. 2022 Sep 27;12(10):1591. doi: 10.3390/jpm12101591 (PMC9605139; doi:10.3390/jpm12101591)
Supplement: Supplementary file 1 [file jpm-12-01591-s001.zip › jpm-1910839-supplementary.pdf]

SUPPLEMENTARY MATERIAL

Table S1. Descriptive analysis of the Spanish population from the Spanish National Health Survey 2017.

**Variables**				
**Age (Years)**	**Overall (n=17777)**	**Men (n=8529)**	**Women (n=9248)**	***P***
Median (IQR)	47 (21)	47 (21)	47 (21)	0.295
**IMC (kg/m2)**	**Overall (n=17273)**	**Men=8368**	**Women=8905**	***P***
Median (IQR)	25.3 (5.9)	26.1 (5.0)	24.2 (6.4)	<0.001
**Prevalence of pain n (%)**	**Overall (n=17773)**	**Men (n=8526)**	**Women (n=9247)**	***p****
Yes	7536 (42.4)	3012 (35.3)	4524 (48.9)*	<0.001
No	10237 (57.6)	5514 (64.7)	4723 (51.1)	
**Degree of pain n (%)**				***p****
None	10237 (57.6)	5514 (64.7)	4723 (51.1)	<0.001
Very light	1390 (7.8)	653 (7.7)	737 (8.0)	
Light	2518 (14.2)	1067 (12.5)	1451 (15.7)	
Moderate	2351 (13.2)	857 (10.1)	1494 (16.2)	
Severe	1062 (6.0)	351 (4.1)	711 (7.7)	
Extreme	215 (1.2)	84 (1.0)	131 (1.4)	
**Pain affect n (%)**	**Overall (n=17773)**	**Men (n=8526)**	**Women (n=9247)**	***p****
Nothing	12388 (69.7)	6473 (75.4)	5915 (64.0)	<0.001
Somewhat	2367 (13.3)	962 (11.3)	1405 (15.2)	
Moderately	1627 (9.2)	592 (6.9)	1035 (11.2)	
Quite a lot	952 (5.4)	335 (3.9)	617 (6.7)	
A lot	439 (2.5)	164 (1.9)	275 (3.0)	
**Pain limitation n (%)**	**Overall (n=17777)**	**Men (n=8529)**	**Women (n=9248)**	***p****
Yes	2383 (13.4)	949 (11.1)	1434 (15.5)*	<0.001
Non	15394 (86.6)	7580 (88.9)	7814 (84.5)	
**Pain medication n (%)**	**Overall (n=17776)**	**Men (n=8529)**	**Women (n=9247)**	***p****
Yes	5491 (30.9)	2020 (23.7)	3471 (37.5)*	<0.001
No	12285 (69.1)	6509 (76.3)	5776 (62.5)	
**Physical Activity Level n (%)**	**Overall (n=17717)**	**Men (n=8489)**	**Women (n=9228)**	***p****
Inactive (PAI=0)	2533 (14.3)	1175 (13.8)	1358 (14.7)	<0.001
Walker (PAI=0)	8067 (45.5)	3390 (39.9)	4667 (50.7))	
Low (PAI=1-15)	2433 (13.7)	1120 (13.2)	1313 (14.2)	
Medium (PAI=16-30)	2456 (13.9)	1341 (15.8)	1115 (12.1)	
High (PAI=31-45)	1472 (8.3)	954 (11.2)	518 (5.6)	
Very High (PAI=+45)	756 (4.3)	509 (6.0)	247 (2.7)	

IQR (Interquartile Range); n (Number of participants); % (Percentage of people in each group); BMI (Body Mass Index); p (p-value from Mann-Whitney U-test); p* (p-value from chi-square test); PAI (Physical Activity Index); Inactive (PAI=0; Declare not to go for a walk for more than 10 minutes at a time); Walker (PAI=0; Declare walking for more than 10 minutes at a time); Low (PAI between 1 and 15); Medium (PAI between 16 and 30); High (PAI between 31 and 45); Very High (PAI greater than 45); * (Significant differences between proportions with p<0.05 in pairwise z-test).

Table S2. Relationship between level of physical activity and prevalence of pain in the Spanish population.

Variable	Physical activity level									
**Overall**										
Pain	Inactive (n=2531)	Walker (n=8066)	Low (n=2433)	Medium (n=2456)	High (n=1472)	Very High (n=756)	x^2	df	p	cc
Yes n (%)	1313 (51.9)a	3648 (45.2)b	986 (40.5)c	863 (35.1)d	469 (31.9)d	237 (31.3)d	280.7	5	<0.001	0.125
No n (%)	1218 (48.1)a	4418 (54.8)b	1447 (59.5)c	1593 (64.9)d	1003 (68.1)d	519 (68.7)d				
**Men**										
	Inactive (n=1174)	Walker (n=3389)	Low (n=1120)	Medium (n=1341)	High (n=954)	Very high (n=509)				
Yes n (%)	537 (45.7)a	1317 (38.9)b	370 (33.0)c	386 (28.8)c,d	257 (26.9)d	133 (26.1)c,d	150.1	5	<0.001	0.132
No n (%)	637 (54.3)a	2072 (61.1)b	750 (67.0)c	955 (71.2)c,d	697 (73.1)d	376 (73.9)c,d				
**Women**										
	Inactive (n=1357)	Walker (n=4677)	Low (n=1313)	Medium (n=1115)	High (n=518)	Very high (n=247)				
Yes n (%)	776 (57.2)a	2331 (51.6)b	616 (46.9)b,c	477 (42.8)c	212 (40.9)c	104 (42.1)b,c	75.4	5	<0.001	0.090
No n (%)	581 (42.8)a	2346 (50.2)b	697 (53.1)b,c	638 (57.2)c	306 (59.1)c	143 (57.9)b,c				

n (participants); % (percentage); PAI (Physical Activity Index); Inactive (PAI=0; Declare not to go for a walk for more than 10 minutes at a time); Walker (PAI=0; Declare walking for more than 10 minutes at a time); Low (PAI between 1 and 15); Medium (PAI between 16 and 30); High (PAI between 31 and 45); Very High (PAI greater than 45); x^2 (Pearson's chi-square); p (p-value); df (degree freedom); cc (Contingency coefficient); abcd (Each subscript letter denotes a subset of PAL whose proportions of prevalence of pain do not differ significantly from each other with p<0.05 from z-test

Table S3. Relationship between level of physical activity and prevalence of pain in the Spanish population.

Variable	Physical activity level									
**Overall**										
Degree of pain	Inactive (n=2531)	Walker (n=8066)	Low (n=2433)	Medium (n=2456)	High (n=1472)	Very High (n=756)	x^2	df	p	cc
Extreme n (%)	86 (3.4)a	87 (1.1)b	14 (0.6)b	18 (0.7)b	5 (0.3)b	6 (0.8)b	566.4	25	<0.001	0.176
Severe n (%)	269 (10.6)a	547 (6.8)b	107 (4.4)c	82 (3.3)c,d	33 (2.2)d	24 (3.2)c,d				
Moderate n (%)	450 (17.8)a	1175 (14.6)b	292 (12.0)c	254 (10.3)c,d	117 (7.9)d	59 (7.8)d				
Light n (%)	364 (14.4)a	1174 (14.6)a	366 (15.0)a	307 (12.5)a	198 (13.5)a	97 (12.8)a				
Very light n (%)	145 (5.7)a	665 (8.2)b	207 (8.5)b	202 (8.2)b	116 (7.9)a,b	51 (6.7)a,b				
None n (%)	1218 (48.1)a	4418 (54.8)b	1447 (59.5)c	1593 (64.9)d	1003 (68.1)d	519 (68.7)d				
**Men**										
	Inactive (n=1174)	Walker (n=3389)	Low (n=1120)	Medium (n=1341)	High (n=954)	Very high (n=509)				
Extreme n (%)	36 (3.1)a	32 (0.9)b	1 (0.1)b	9 (0.7)b	3 (0.3)b	3 (0.6)b	297.5	25	<0.001	0.184
Severe n (%)	89 (7.6)a	175 (5.2)b	35 (3.1)b,c	30 (2.2)c	14 (1.5)c	8 (1.6)c				
Moderate n (%)	180 (15.3)a	393 (11.6)b	84 (7.5)c	104 (7.8)c	65 (6.8)c	28 (5.5)c				
Light n (%)	166 (14.1)a	437 (12.9)a	147 (13.1)a	144 (10.7)a	111 (11.6)a	56 (11.0)a				
Very light n (%)	66 (5.6)a	280 (8.3)b	103 (9.2)b	99 (7.4)a,b	64 (6.7)a,b	38 (7.5)a,b				
None n (%)	637 (54.3)a	2072 (61.1)b	750 (67.0)c	955 (71.2)c,d	697 (73.1)d	376 (73.9)c,d				
**Women**										
	Inactive (n=1357)	Walker (n=4677)	Low (n=1313)	Medium (n=1115)	High (n=518)	Very High (n=247)				
Extreme n (%)	49 (3.6)a	55 (1.2)c	13 (1.0)b	9 (0.8)b	2 (0.4)b	3 (1.2)a,b	231.9	25	<0.001	0.157
Severe n (%)	180 (13.3)a	372 (8.0)b	72 (5.5)c	52 (4.7)c	19 (3.7)c	16 (6.5)b,c				
Moderate n (%)	270 (19.9)a	782 (16.7)a,b	208 (15.8)a,b	150 (13.5)b,c	52 (10.0)c	31 (12.6)a,b,c				
Light n (%)	198 (14.6)a	737 (15.8)a	219 (16.7)a	163 (14.6)a	87 (16.8)a	41 (16.6)a				
Very light n (%)	79 (5.8)a	385 (8.2)a,b	104 (7.9)a,b	103 (9.2)b	52 (10.0)b	13 (5.3)a,b				
None n (%)	581 (42.8)a	2346 (50.2)b	697 (53.1)a,b	638 (57.2)c	306 (59.1)c	143 (57.9)b,c				

n (participants); % (percentage); PAI (Physical Activity Index); Inactive (PAI=0; Declare not to go for a walk for more than 10 minutes at a time); Walker (PAI=0; Declare walking for more than 10 minutes at a time); Low (PAI between 1 and 15); Medium (PAI between 16 and 30); High (PAI between 31 and 45); Very High (PAI greater than 45); x^2 (Pearson's chi-square); p (p-value); df (degree freedom); cc (Contingency coefficient); abcd (Each subscript letter denotes a subset of PAL whose proportions of each prevalence of degree of pain do not differ significantly from each other with $p<0.05$ from z-test.

Table S4. Relationship between level of physical activity and prevalence of pain in the Spanish population.

Overall										
Variable	Physical activity level									
Pain affect	Inactive (n=2531)	Walker (n=8067)	Low (n=2433)	Medium (n=2456)	High (n=1472)	Very High (n=756)	x^2	df	p	cc
Nothing n(%)	1463 (57.8)a	5402 (67.0)b	1777 (73.0)c	1914 (77.9)d	1177 (80.0)d	608 (80.4)d				
Somewhat n(%)	356 (14.1)a,b	1138 (14.1)b	333 (13.7)a,b	278 (11.3)a	172 (11.7)a,b	83 (11.0)a,b				
Moderately n(%)	309 (12.2)a	855 (10.6)a	199 (8.2)b	155 (6.3)b,c	75 (5.1)c	30 (4.0)c	603.3	20	<0.001	0.181
Quite a lot n(%)	247 (9.8)a	479 (6.0)b	89 (3.7)c	78 (3.2)c	33 (2.2)c	26 (3.4)b,c				
A lot n(%)	156 (6.2)a	192 (2.4)b	35 (1.4)b,c	31 (1.3)c	15 (1.0)c	9 (1.2)b,c				
Men										
	Inactive (n=1174)	Walker (n=3390)	Low (n=1120)	Medium (n=1341)	High (n=954)	Very High (n=509)				
Nothing n(%)	742 (63.2)a	2459 (72.5)b	892 (79.6)c	1119 (83.4)c	798 (83.6)c	433 (85.1)c				
Somewhat n(%)	151 (12.9)a	430 (12.8)a	126 (11.3)a,b	113 (8.4)b	95 (10.0)a,b	42 (8.3)a,b				
Moderately n(%)	123 (10.5)a	282 (8.3)a,b	66 (5.9)b,c	64 (4.8)c	38 (4.0)c	16 (3.1)c	321.5	20	<0.001	0.191
Quite a lot n(%)	97 (8.3)a	150 (4.4)b	22 (2.0)c	36 (2.7)b,c	16 (1.7)c	15 (2.9)b,c				
A lot n(%)	61 (5.2)a	69 (2.0)b	14 (1.3)b,c	9 (0.7)c	7 (0.7)b,c	3 (0.6)b,c				
Women										
	Inactive (n=1357)	Walker (n=4677)	Low (n=1313)	Medium (n=1115)	High (n=518)	Very High (n=247)				
Nothing n(%)	721 (53.1)a	2943 (62.9)b	885 (67.4)c	795 (71.3)c	379 (73.2)c	175 (70.9)b,c				
Somewhat n(%)	205 (15.1)a	708 (15.1)a	207 (15.8)a	165 (14.8)a	77 (14.9)a	41 (16.6)a				
Moderately n(%)	186 (13.7)a	573 (12.3)a	133 (10.1)a,b	91 (8.2)c	37 (7.1)b	14 (5.7)b	245.3	20	<0.001	0.161
Quite a lot n(%)	150 (11.1)a	330 (7.1)b	67 (5.1)b,c	42 (3.8)c	17 (3.3)c	11 (4.5)b,c				
A lot n(%)	95 (7.0)a	123 (2.6)b	21 (1.6)b	22 (2.0)b	8 (1.5)b	6 (2.4)a,b				

n (participants); % (percentage); PAI (Physical Activity Index); Inactive (PAI=0; Declare not to go for a walk for more than 10 minutes at a time); Walker (PAI=0; Declare walking for more than 10 minutes at a time); Low (PAI between 1 and 15); Medium (PAI between 16 and 30); High (PAI between 31 and 45); Very High (PAI greater than 45); x^2 (P-value from chi-square test); abcd (Each subscript letter denotes a subset of PAL whose proportions of each prevalence of pain affect do not differ significantly from each other with p<0.05 from z-test).

Table S5. Relationship between level of physical activity and suffering limitations to perform daily activities due to pain in the Spanish population of the ENSE 2017.

Overall										
Variable	Physical activity level									
Limitation due to pain	Inactive (n=2533)	Walker (n=8067)	Low (n=2433)	Medium (n=2456)	High (n=1472)	Very High (n=756)	x^2	df	p	cc
Yes n (%)	545 (21.5)a	1136 (14.1)b	261 (10.7)c	241 (9.8)c	125 (8.5)c	71 (9.4)c	232.5	6	<0.001	0.114
No n (%)	1988 (78.5)a	6931 (85.9)b	2172 (89.3)c	2215 (90.2)c	1347 (91.5)c	685 (90.6)c				
Men										
	Inactive (n=1174)	Walker (n=3389)	Low (n=1120)	Medium (n=1341)	High (n=954)	Very high (n=509)				
Yes n (%)	221 (18.8)a	398 (11.7)b	101 (9.0)b,c	118 (8.8)b,c	67 (7.0)c	41 (8.1)b,c	105.5	6	<0.001	0.110
No n (%)	954 (81.2)a	2992 (88.3)b	1019 (91.0)b,c	1223 (91.2)b,c	887 (93.0)c	468 (91.9)b,c				
Women										
	Inactive (n=1357)	Walker (n=4677)	Low (n=1313)	Medium (n=1115)	High (n=518)	Very high (n=247)				
Yes n (%)	324 (23.9)a	738 (15.8)b	160 (12.2)c	123 (11.0)c	58 (11.2)b,c	30 (12.1)b,c	111.8	6	<0.001	0.109
No n (%)	1034 (76.1)a	3939 (84.2)b	1153 (87.8)c	992 (89.0)c	460 (88.8)b,c	217 (87.9)b,c				

n (participants); % (percentage); PAI (Physical Activity Index); Inactive (PAI=0; Declare not to go for a walk for more than 10 minutes at a time); Walker (PAI=0; Declare walking for more than 10 minutes at a time); Low (PAI between 1 and 15); Medium (PAI between 16 and 30); High (PAI between 31 and 45); Very High (PAI greater than 45); x^2 (Pearson's chi-square); p (p-value from chi-square test); df (Degree freedom); cc (Contingency coefficient); abcd (Each subscript letter denotes a subset of PAL whose proportions of prevalence of limitation due to pain do not differ significantly from each other with p<0.05 from z-test.

Table S6. Relationship between level of physical activity and the use of pain medication in the Spanish population in the ENSE 2017.

Variable	Physical activity level									
**Overall**										
Pain medication	Inactive (n=2533)	Walker (n=8066)	Low (n=2433)	Medium (n=2456)	High (n=1472)	Very High (n=756)	x2	df	p	cc
Yes n (%)	939 (37.1)a	2723 (33.8)b	721 (29.6)c	577 (23.5)d	337 (22.9)d	184 (24.3)c,d	200.3	5	<0.001	0.106
No n (%)	1594 (62.9)a	5343 (66.2)b	1712 (70.4)c	1879 (76.5)d	1135 (77.1)d	572 (75.7)c,d				
**Men**										
	Inactive (n=1175)	Walker (n=3390)	Low (n=1120)	Medium (n=1341)	High (n=954)	Very High (n=509)				
Yes n (%)	351 (29.9)a	887 (26.2)a,b	256 (22.9)b,c	239 (17.8)d	176 (18.4)c,d	104 (20.4)b,c,d	80.9	5	<0.001	0.096
No n (%)	824 (70.1)a	2503 (73.8)a,b	864 (77.1)b,c	1102 (82.2)d	778 (81.6)c,d	405 (79.6)b,c,d				
**Women**										
	Inactive (n=1358)	Walker (n=4676)	Low (n=1313)	Medium (n=1115)	High (n=518)	Very High (n=247)				
Yes n (%)	588 (43.3)a	1836 (39.3)a,b	465 (35.4)b,c	338 (30.3)c	161 (31.1)c	80 (32.4)b,c	64.5	5	<0.001	0.083
No n (%)	770 (56.7)a	2840 (60.7)a,b	848 (64.6)b,c	777 (69.7)c	357 (68.9)c	167 (67.6)b,c				

n (participants); % (percentage); PAI (Physical Activity Index); Inactive (PAI=0; Declare not to go for a walk for more than 10 minutes at a time); Walker (PAI=0; Declare walking for more than 10 minutes at a time); Low (PAI between 1 and 15); Medium (PAI between 16 and 30); High (PAI between 31 and 45); Very High (PAI greater than 45); p (P-value from chi-square test); abcd (Each subscript letter denotes a subset of PAL whose proportions of prevalence of use of pain medication do not differ significantly from each other with $p<0.05$ from z-test)
